# Cervical Erector Spinae Plane Block in a Patient with Failed Neck Surgery Syndrome: A Case Report and Literature Review

**DOI:** 10.5812/aapm-161511

**Published:** 2025-05-20

**Authors:** Poupak Rahimzadeh, Sara Saadat, Sajede Salehi

**Affiliations:** 1 Pain Research Center, Department of Anesthesiology and Pain Medicine, School of Medicine, Iran University of Medical Sciences, Tehran, Iran; 2 Department of Anesthesiology and Pain Medicine, Iran University of Medical Sciences, Tehran, Iran; 3 Department of Anesthesia and Pain Medicine, Baqiyatallah University of Medical Sciences, Tehran, Iran

**Keywords:** Cervical Erector Spinae Plane Block, Failed Neck Surgery Syndrome, Numerical Rating Scale, Neck Disability Index, Posterior Spinal Fusion

## Abstract

**Introduction:**

Failed neck surgery syndrome (FNSS) following posterior cervical spine fusion (PSF) presents a considerable challenge in pain management.

**Objective:**

This study aims to report the potential utility of the cervical erector spinae plane block (ESPB) as an influential adjunctive therapy in a patient with FNSS who was refractory to medical therapy and unable to undergo spinal cord stimulation (SCS) due to financial constraints.

**Methods:**

This case report details a 74-year-old male with persistent, debilitating axial neck pain lasting one year following PSF, without neural or motor deficits, and with device failure ruled out. Given the risks of cervical epidural steroid injection in this patient, a fluoroscopically guided bilateral cervical ESPB at the C7 level was administered for temporary pain relief.

**Result:**

This technique was associated with substantial pain relief and improved functional outcomes, as demonstrated by reductions in the Numerical Rating Scale (NRS) and Neck Disability Index (NDI) scores.

**Conclusion:**

This case underscores the potential utility of the cervical ESPB as an effective adjunctive therapy for managing pain in FNSS, providing a minimally invasive alternative to conventional treatment approaches.

## 1. Introduction

Failed neck surgery syndrome (FNSS) is characterized by persistent or recurrent pain following spinal surgical interventions (1). Managing cervical FNSS poses a particular challenge; yet, one established costly modality for pain management is cervical spinal cord stimulation (SCS) (2), which may not be a viable option for all patients. Consequently, less invasive interventional techniques, such as epidural steroid injections (ESIs), are frequently employed for temporary symptom relief (3). However, FNSS patients present unique anatomical challenges for cervical epidural steroid injections (CESIs) due to dural thinning, disruption, and epidural fibrosis, leading to an increased risk of dural tear and cerebrospinal fluid (CSF) leaks (4-6). Given these considerations, emerging alternatives, such as the erector spinae plane block (ESPB), have shown promise in certain clinical scenarios (3).

The ESPB has been extensively utilized for thoracic and lumbar pain management, yet its efficacy in treating cervical pain remains less well-documented. This case report highlights the successful application of a fluoroscopy-guided cervical ESPB for managing axial neck pain in a patient with FNSS, underscoring its potential role as an effective interventional strategy. This study aims to report the potential utility of the cervical ESPB as an influential adjunctive therapy in a patient with FNSS who was refractory to medical therapy and unable to undergo SCS due to financial constraints.

## 2. Case Presentation

We present the case of a 74-year-old male with persistent axial neck pain following posterior cervical spine fusion (PSF) from the first cervical vertebra (C1) to the fifth cervical vertebra (C7), persisting for one year. It is worth noting that the patient underwent cervical PSF 18 months ago; however, the pain developed gradually afterward and has worsened over the past year. The patient described his pain as constant, with intermittent sharp, debilitating exacerbations triggered by movement. Over time, his symptoms progressively worsened, and four months prior to presentation, the pain had become intolerable, significantly affecting his quality of life and causing distress to his family. His neurosurgeon advised against revision surgery. However, the pain proved refractory to high-dose NSAIDs, muscle relaxants, and even opioid therapy, and the patient was unable to tolerate physical therapy. Consequently, the neurosurgeon referred him to our pain clinic for further management.

At the time of evaluation, the patient reported a Numerical Rating Scale (NRS) score of 10 out of 10 (on a scale of 0 = no pain, to 10 = the most severe pain experienced), and a Neck Disability Index (NDI; 0 - 5 = mild, 6 - 15 = moderate, 16 - 25 = severe, > 26 = very severe) score of 35, while he was on a high dose of NSAIDs, pregabalin, and opium, indicating severe pain and functional impairment. On physical examination, he exhibited restricted cervical spine mobility and marked tenderness over the paraspinal cervical muscles. However, no neurological deficits were noted, and deep tendon reflexes were intact. As previously noted, imaging studies — including plain radiography and cervical spine MRI — demonstrated no evidence of device displacement or spinal cord compression. The only finding was neural foraminal narrowing secondary to degenerative changes, which was not associated with a neural deficit. Given the high initial cost of SCS, the patient declined this intervention, necessitating an alternative pain management approach to provide temporary relief until he could consider SCS.

Considering the extensive surgical manipulation in the posterior cervical region and the high likelihood of dural integrity disruption, and after consultation with the patient and his family and with their permission, we opted for a bilateral cervical ESPB as an interventional pain management strategy.

### 2.1. Procedure

On the 29th of July, the patient was referred to the operating room and positioned prone with a pillow placed under the chest to achieve cervical spine flexion, adjusted to his tolerance. Standard monitoring, including electrocardiography (ECG), noninvasive blood pressure measurement, and pulse oximetry, was applied. The posterior cervical and upper thoracic regions were aseptically prepared and draped. Under fluoroscopic guidance, an anteroposterior (AP) view was obtained to visualize and confirm the C7 vertebral body. The transverse process of C7 was identified and marked. A 22-gauge, 90-mm spinal needle (Disposable Spinal Needle, Dr. Japan Co., Ltd., Tokyo, Japan) was advanced toward the tip of the C7 transverse process under fluoroscopic guidance. Once bony contact was achieved and negative aspiration was confirmed, 2 mL of Visipaque contrast (VISIPAQUE 320 mg I/mL, 50 mL vial, GE Healthcare AS, Oslo, Norway) was injected to verify contrast spread within the erector spinae muscle plane under coaxial fluoroscopic visualization. Following confirmation of appropriate contrast distribution, 20 mL of 0.25% ropivacaine (Ropivacaine Hydrochloride, Bioindustria L.I.M., 5 mg/mL, Italy) combined with 20 mg of triamcinolone (Triamcinolone, CBCORT 40 mg/1 mL, Chandra Bhagat Pharma, India) was administered. The same procedure was then performed on the contralateral side. Upon completion, the needle was removed, and the patient was transferred to the recovery room for post-procedural monitoring. The patient was observed in the recovery unit for two hours, during which vital signs remained stable, and no immediate complications were noted. He was subsequently discharged with instructions for follow-up assessment. He was advised to continue pregabalin and opium and cease consuming NSAIDs.

## 3. Result

One week after the procedure, the patient reported a significant reduction in pain, with an NRS score of 0 and an NDI score of 8. He also experienced a marked improvement in quality of life, with greater ease in performing daily activities. At the two-week follow-up, the patient reported mild residual pain, with an NRS score of 2 and an NDI score of 9. Given the sustained but gradually diminishing pain relief, the procedure was repeated at the two-week mark to extend its therapeutic benefits. At the one-month follow-up after the second procedure, he expressed overall satisfaction with the treatment outcome and was able to resume most of his routine activities without significant limitations (NRS = 2 and NDI = 11). He had ceased pregabalin on his own and was tapering opium so that he needed a low dose of opium. This positive response persisted over subsequent three-month and five-month follow-ups with a 70% reduction of NRS and NDI after the second procedure, further supporting the efficacy of fluoroscopy-guided cervical ESPB in managing chronic post-surgical neck pain. No complications or adverse effects were observed throughout the follow-up period.

## 4. Discussion

The erector spinae muscles consist of three longitudinal muscle columns — spinalis cervicis, longissimus cervicis, and iliocostalis cervicis — that run bilaterally along the vertebral column in the cervical region (7). Since its introduction, ESPB has been widely applied in the management of both acute and chronic pain conditions (8-11). Although its precise mechanism of action remains incompletely understood, it is hypothesized that local anesthetic spreads within the fascial plane, leading to blockade of the posterior rami of the spinal nerves and, potentially, anterior extension into the paravertebral space, blocking both dorsal and ventral rami of spinal nerves (12), making it a promising alternative to epidural or paravertebral blocks (13, 14). This mechanism provides effective analgesia while avoiding deeper, more invasive procedures such as CESI (15, 16).

Notably, due to both the surgical manipulation of the cervical region and the presence of hardware, performing an image-guided medial branch block (MBB) presented significant technical challenges. Additionally, the required positioning and repeated needle manipulations would have been poorly tolerated by this patient. Therefore, we determined this approach not to be considered in this case.

Furthermore, in this case, ultrasound-guided ESPB was not our preferred approach due to the altered cervical anatomy from prior surgical manipulation. We instead utilized fluoroscopic guidance to ensure precise needle placement within the fascial plane, thereby minimizing the risk of intrathecal injection — a critical safety consideration in patients with a history of PSF. Studies suggest that approximately 3 - 5 mL of local anesthetic is required per dermatome to achieve effective blockade (17). Based on this evidence, we administered 20 mL of local anesthetic bilaterally to achieve sufficient diffusion and pain relief. This case highlights the successful management of axial cervical pain in a patient with FNSS who was refractory to medical therapy and unable to undergo SCS due to financial constraints. Following two bilateral cervical ESPB procedures, the patient experienced significant and long-lasting pain reduction. Our findings align with the study by Hong and Huh, which demonstrated that high thoracic ESPB provides pain relief comparable to CESI in patients with cervical radicular pain (18). The favorable outcomes observed in this patient suggest that fluoroscopy-guided cervical ESPB is a viable, minimally invasive option for pain management in FNSS, offering both analgesia and functional improvement.

### 4.1. Conclusions

In this single case, fluoroscopy-guided cervical ESPB proves to be a promising and minimally invasive option for managing axial neck pain due to FNSS. The placebo effect and natural fluctuations in pain cannot be fully excluded. However, the dramatic improvement in objective scores (NRS, NDI) and the temporal relationship to the ESPB make a strong argument for its effectiveness in this scenario and suggest that cervical ESPB may be a valuable alternative to more invasive interventions, such as SCS or CESIs. This warrants further investigation with larger sample sizes and controlled trials to validate its broader application in clinical practice.

## Data Availability

The dataset presented in the study is available on request from the corresponding author during submission or after publication.
